# Controlling the terminal layer atom of InTe for enhanced electrochemical oxygen evolution reaction and hydrogen evolution reaction performance†

**DOI:** 10.1039/d3na00142c

**Published:** 2023-03-22

**Authors:** Jie Wu, Zhiyu Shao, Beining Zheng, Yuan Zhang, Xiangdong Yao, Keke Huang, Shouhua Feng

**Affiliations:** a State Key Laboratory of Inorganic Synthesis and Preparative Chemistry and Jilin Provincial International Cooperation Key Laboratory of Advanced Inorganic Solid Functional Materials, College of Chemistry, Jilin University Qianjin Street 2699 Changchun 130012 China shfeng@jlu.edu.cn

## Abstract

Herein, we report the method of molecular-beam-epitaxial growth (MBE) for precisely regulating the terminal surface with different exposed atoms on indium telluride (InTe) and studied the electrocatalytic performances toward hydrogen evolution reaction (HER) and oxygen evolution reaction (OER). The improved performances result from the exposed In or Te atoms cluster, which affects the conductivity and active sites. This work provides insights into the comprehensive electrochemical attributes of layered indium chalcogenides and exhibits a new route for catalyst synthesis.

## Abstract 2

The terminal layer atoms of InTe was regulated *via* molecular-beam-epitaxial growth to achieve enhanced electrochemical oxygen evolution reaction and hydrogen evolution reaction performance.

## Graphical abstract

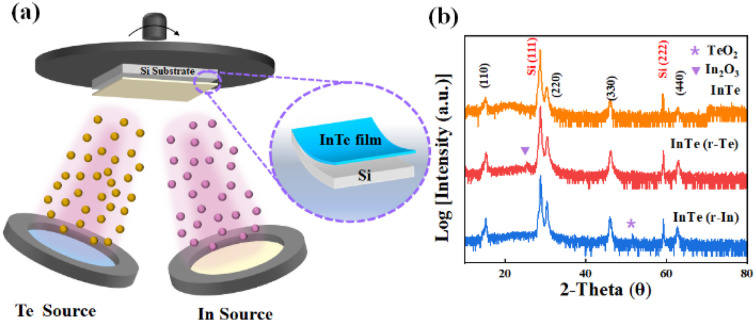

Facing the energy crisis and the unbearable environmental pressure on human beings, it is urgent to develop new clean energy sources.^1–3^ Water splitting is considered as a very promising alternative method to fossil fuels because of its cleanliness and high energy density of the products.^4^ At present, electrocatalysis is mainly focused on the regulation of intrinsic characteristics and the design of active sites.^5,6^ On the one hand, the intrinsic characteristics of the catalyst was further enhanced by changing the atomic arrangement and electronic structure of the catalyst through heteroatom doping, defect construction, and composite engineering.^7–9^ On the other hand, the abundance of active sites can be increased by surface reconstruction, increasing the specific surface area, and constructing surface unsaturated sites.^10–12^ However, identifying the true impact factors of catalytic reaction is difficult because of the complex structure of the catalyst for both mechanisms. Therefore, a simple reaction model needs to be proposed to study the active sites of the reaction.

Two-dimensional (2D) materials are considered as ideal models for studying the growth mechanism and catalytic reaction mechanism, which have been widely used in the fields of photonics, electronics, catalysis, and energy storage.^13–15^ For instance, 2D group III monochalcogenides (MXs, M = Ga and In; X = S, Se, and Te) monolayers were theoretically predicted to exhibit fascinating magneto-optical effects and electronic properties.^16^ Besides, 2D InSe monolayer exhibited better environmental stability than other similar materials.^17^ However, the development of precisely controlled synthesis of 2D materials is still progressing. Traditional methods, such as hydrothermal synthesis, cannot ensure the purity and tend to form defects and separate phases with various chemical compositions. The CVD growth of 2D materials is suffers from a lack of spatial distribution controllability because the volatile source sublimes unevenly along the same diffusion path.^18^ Therefore, it is of great significance to develop a scalable and highly controlled approach for synthesizing 2D materials with homogeneous distribution and adjustable exposed surfaces.

In this work, molecular-beam-epitaxial growth (MBE) was used to prepare InTe samples; In-rich InTe and Te-rich InTe were realized by annealing under indium and tellurium atmosphere, respectively. X-ray photoelectron spectroscopy (XPS) and atomic force microscopy (AFM) indicated that the enrichment of elements at the atomic level can be effectively regulated, which not only leads to vacancies but also gives rise to the exposed unsaturated sites on the surface of the InTe samples. The electrochemical properties toward hydrogen evolution reaction (HER) and oxygen evolution reaction (OER) were also enhanced with different enriched elements. The study of the electrochemical reaction mechanism and the preparation of new catalysts is of great significance.

The synthetic scheme of InTe films is illustrated in Fig. 1a. InTe films were deposited on Si (111) substrate in an ultrahigh vacuum chamber with a base pressure of 2 × 10^−9^ torr. Initially, Si (111) substrate was cleaned by the standard RCA process and degassed at 800 °C for 30 min. The substrate was then cooled to 370 °C for InTe growth. Solid In and Te effusion cells were used with a beam equivalent pressure (BEP) flux ratio of Te : In of approximately 5 : 2. After the deposition of InTe films, In-rich InTe (InTe (r-In)) and Te-rich InTe (InTe(r-Te)) were capped by indium and tellurium flux at 370 °C, respectively.^19^

**Fig. 1:**
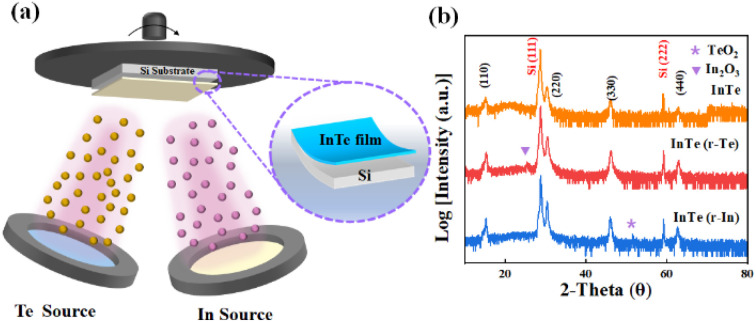
(a) Schematic illustration of the molecular beam epitaxy system used for the synthesis of InTe thin films, (b) XRD analysis of InTe thin films.

The XRD analysis of InTe thin films is shown in Fig. 1b, which demonstrates that the films grown on Si (111) are epitaxial and elongate along the polar 〈110〉 axis. Additional characteristic peaks can be indexed to TeO_*x*_ (PDF#71-0508) and In_2_O_3_ (PDF# 71-2194) in InTe (r-In) and InTe (r-Te), respectively, suggesting that the disordered arrangement of terminal atoms can affect the surface composition.^20–22^ Coordination unsaturation at the atomic level leads to the formation of the corresponding oxide when the samples are exposed to air. In addition, the XRD patterns of temperature-controlled samples are also shown in Fig. S1 (ESI†), demonstrating good crystallization and the characteristics of epitaxial growth.

The morphologies of the materials were observed by scanning electron microscopy (SEM) and transmission electron microscopy (TEM). As shown in Fig. 2a and Fig. S2a (ESI†), the InTe film presents a uniform and compact structure with the island growth mode. Fig. 2b and c and Fig. S2b and c (ESI†) show the process flow of InTe modified in tellurium and indium flux, where their layered structures showed a smooth surface of InTe converted to a rougher surface and appeared flaky and linear, respectively, which is related to the solid phase epitaxy mode and further leads to the formation of unsaturated sites. Roughness and surface aggregation of components can be more clearly observed by AFM in Fig. 2d–f. The HRTEM images show the layered flakes of InTe with a high degree of crystallinity in Fig. 2g, and the as-observed lattice fringes of 0.29 nm correspond to the (220) lattice planes of InTe. The aggregated components with lattice fringes of 0.30 and 0.33 nm of InTe (112) planes and (211) planes grow from the silicon, as seen in Fig. 2h and i, respectively. The inset images in the left of Fig. 2h and i are the selected area electron diffraction (SAED) patterns, and the (002), (004), (023), (202), and (200) lattice planes are indexed to InTe, respectively.^23^

**Fig. 2:**
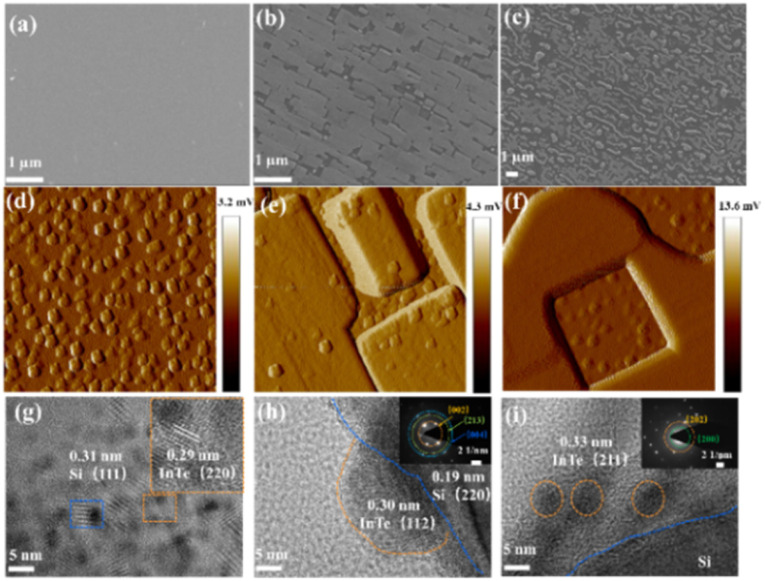
The SEM images of (a) InTe, (b) InTe (r-Te), and (c) InTe (r-In). The AFM images of (d) InTe, (e) InTe (r-Te), and (f) InTe (r-In). The HRTEM images and SAED pattern of (g) InTe, (h) InTe (r-Te), and (i) InTe (r-In).

X-ray photoelectron spectroscopy (XPS) was carried out to further investigate the valence state and surface composition (Fig. 3). The survey spectra (Fig. 3a) show the existence of Te, O, In, and C elements on the surface of all the prepared catalysts. As shown in Fig. 3b, the deconvolution of In 3d revealed spin–orbit split doublets located at 452.3, 452.5 eV and 444.6, 445.3.0 eV, which is attributed to In (0) and In–Te, respectively.^24^ The obvious increase in the content of In (0) in InTe (r-Te) and InTe (r-In) is due to the enrichment of surface elements in Table S1 (ESI†). It is worth noting that Te-rich InTe also caused the unsaturated coordination of the surface. The two pairs of peaks located at 572.7, 583.3 eV and 576.7, 586.7 eV can be observed in the Te 3d spectrum and are attributed to In–Te and Te (0), respectively.^25,26^ In the O 1s XPS spectrum (Fig. 3d), the peaks located at 529.5 eV (O_I_), 532.4 eV (O_II_), and 532.5 eV (O_III_) confirmed the presence of adsorbed oxygen, oxyhydroxide, and oxide, which can be assigned to the M–O bonds, M–O–H bonds, and H_2_O, respectively.^27^ Comparing the core-level XPS spectra of O of InTe, the characteristic peaks of oxide can be observed only in InTe (r-Te) and InTe (r-In), which further indicated the presence of an unsaturated site and its susceptibility to oxidation when exposed to air.

**Fig. 3:**
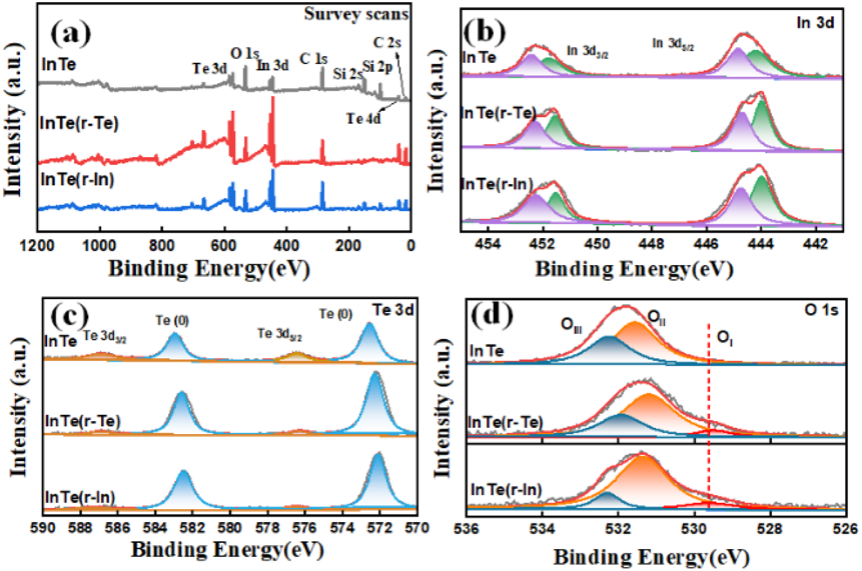
(a) Survey spectra, high-resolution XPS spectra of (b) the In 3d region, (c) the Te 3d region, and (d) O 1s region for InTe, InTe (r-Te), and InTe (r-In).

Electrochemical tests were performed to evaluate the catalytic performance toward the hydrogen evolution reaction (HER) and oxygen evolution reaction (OER) on a typical three-electrode system (see the Experimental section), as shown in the OER and HER linear sweep voltammetry (LSV) curves in Fig. 4a and c, respectively. The surface enriched samples exhibit a much lower overpotential and a larger current density. Specifically, InTe (r-Te) exhibits a better OER catalytic activity and InTe (r-In) exhibits a better HER catalytic activity than the counterparts, respectively, suggesting that the surface unsaturated sites caused by the surface atomic species growth mode can enhance the catalytic performance. The Tafel slope is a principal kinetic parameter to assess the electrochemical behaviors. As shown in Fig. 4b and d, InTe (r-In) delivers a small Tafel slope toward both OER and HER. The much lower Tafel slope value of InTe (r-In) suggests more favorable reaction kinetics that leads to a more rapid reactive rate. The HER limiting step of InTe samples are identified as Volmer adsorption step as the value is close to 120 mV dec^−1^.H_3_O + e^−^ = H_ads_ + H_2_O

**Fig. 4:**
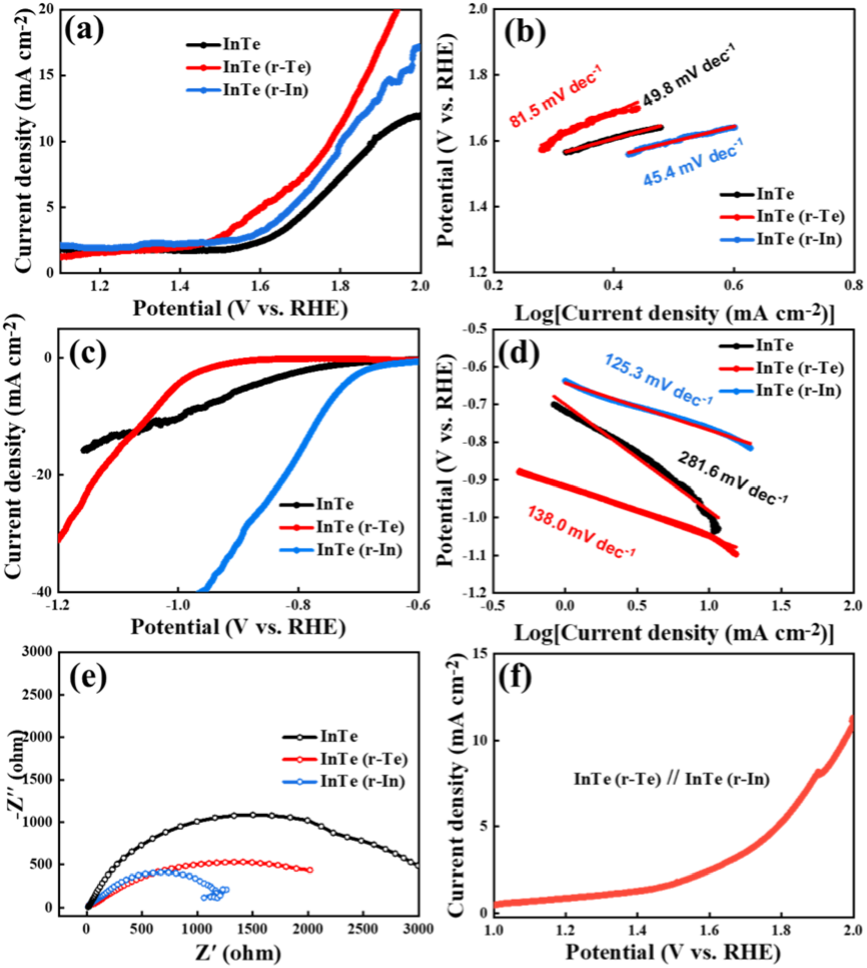
(a and b) OER polarization curves and Tafel plots, (c and d) HER polarization curves and Tafel plots, (e) Nyquist plots, and (f) water splitting curves composed of InTe (r-Te) and InTe (r-In).

Therefore, In-enriched is beneficial for the adsorption of the reaction.^28^

Electrochemical impedance spectroscopy (EIS) was further conducted to explore the charge transfer kinetics.^29^ In the Nyquist plots in Fig. 4e and a smaller semicircle radius corresponds to lower charge transfer resistance (*R*_ct_). InTe (r-In) exhibits the smallest radius among all the samples, suggesting the lowest *R*_ct_ and the fastest electrocatalytic reaction kinetics. Based on the different catalytic activities of the InTe (r-Te) and InTe (r-In), a water splitting device was assembled through InTe (r-Te) at the anode and InTe (r-In) at the cathode. As shown in Fig. 4f, the electrocatalyst reached a cell voltage of about 1.96 V at 10 mA cm^−2^. The electrochemical performance of InTe through MBE on Si (111) proved to be an effective pathway toward the rational and controllable synthesis of bifunctional electrocatalysts for energy applications. Subsequently, we examined the valence and composition changes of InTe catalysts after OER or HER testing by XPS in Fig. S4 (ESI†). The weak signal indicates that the samples are very thin, and the XPS signals are not obvious after reaction. In addition, In showed a relatively strong XPS signal in InTe (r-In) samples, illustrating the surface enrichment of In.

The active electrochemical surface area (ECSA) is shown in Fig. S5 (ESI†). The electrochemical double-layer capacitance (*C*_dl_) was first measured by the CV test with different scan rates. InTe (r-In) and InTe (r-Te) show greater *C*_dl_ and ECSA values, indicating a larger surface area of the catalytic activity.^30^ Besides, the TEM images after the OER and HER are provided in Fig. S6 and S7 (ESI†). The samples still retain the morphology of the ultrathin sheet, and the amount of crystallization exhibits a slight decrease.

## Conclusion

In summary, Te-rich InTe and In-rich InTe nanosheet on Si have been successfully fabricated as electrocatalysts for OER, HER, and water splitting. Although such catalysts have exhibited a less impressive performance, a new avenue with precise regulation at the atomic level is still provided. These fundamental findings provide insights into the comprehensive electrochemical attributes of layered indium chalcogenides, which can be useful for future applications.

## Conflicts of interest

There are no conflicts to declare.

## Supplementary Material

NA-005-D3NA00142C-s001
