# Supplementary material for: Controlling the terminal layer atom of InTe for enhanced electrochemical oxygen evolution reaction and hydrogen evolution reaction performance
Source: Nanoscale Adv. 2023 Mar 22;5(9):2418–21. doi: 10.1039/d3na00142c (PMC10153078; doi:10.1039/d3na00142c)
Supplement: NA-005-D3NA00142C-s001 [file NA-005-D3NA00142C-s001.pdf]

Electronic Supplementary Material (ESI) for Nanoscale Advances.
This journal is © The Royal Society of Chemistry 2023

Supporting Information

Controlling the terminal layer atom of InTe for enhanced electrochemical oxygen evolution reaction and hydrogen evolution reaction performance

Jie Wu,[a] ‡ Zhiyu Shao, [a] ‡ Beining Zheng, [a] Yuan Zhang, [a] Xiangdong Yao, [a] Keke Huang, [a] and Shouhua Feng[a] *

Experimental Section

Materials

Tellurium and Indium of 99.9999% purity were obtained from Alfa Aesar. Potassium hydroxide and sulfuric acid were purchased from Sigma-Aldrich, Singapore. Counter platinum and reference Ag/AgCl, Hg/HgO electrodes were obtained from CH Instruments, Texas, USA.

Materials characterizations

Scanning electron microscopy (SEM) was performed using a JEOL 7800F field-emission SEM (JEOL, Japan) at the voltage of 3 kV. Besides, Energy dispersive X-ray spectroscopy (EDS) was conducted at an acceleration voltage of 20 kV. X-ray powder diffraction was conducted on Bruker D8 Discoverer (Bruker, Germany) powder diffractometer, and Bragg-Brentano geometry was focused using CuKα radiation (λ=0.15418 nm, U= 40 kV, I=40 mA) at room temperature. The data were scanned over an

angular range of 10–80° (2θ) with a step size of 0.016° (2θ). Data evaluation was performed in the software package EVA. High resolution transmission electron microscopy (HR-TEM) was conducted by using an EFTEM JEOL 2200FS microscope (JEOL, Japan) at the accelerated voltage of 200 KeV. Element maps were obtained using the SDD detector X-MaxN 80 TS from Oxford Instruments (England). X-ray photoelectron spectroscopy (XPS) was performed by monochromatic Mg Kα source (SPECS, Germany) at 1253 eV and a multi-channel energy analyzer (SPECS Phoibos 100 MCD-5). Wide scan and high-resolution core level spectra for analysis were collected and C 1s at 284.5 eV was used for calibration.

Linear sweep voltammetry (LSV) was conducted in a three-electrode system where modified glassy carbon, Ag/AgCl or Hg/HgO, and Pt electrode as working, reference, and counter electrode.

Electrocatalytic measurements towards hydrogen evolution, oxygen evolution and water splitting were performed. 0.5 M H_2SO_4 was used for HER measurements and 1.0 M KOH was used for OER and water splitting.

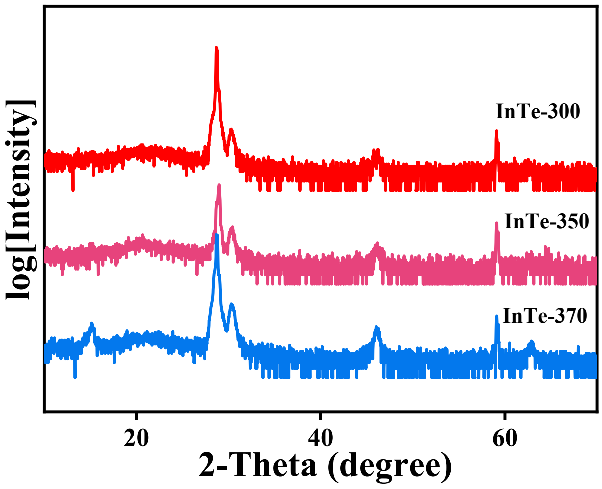

Fig. S1 XRD analysis of InTe thin films with different temperature.

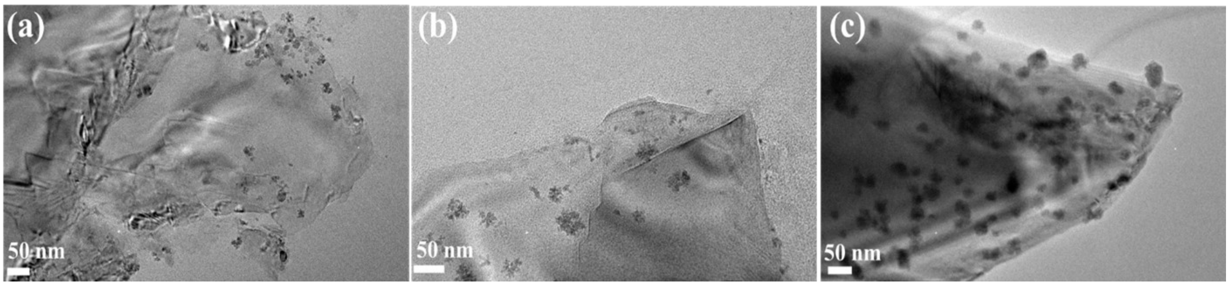

Fig.S2 The TEM images of (a) InTe, (b) InTe (r-Te), and (c) InTe (r-In).

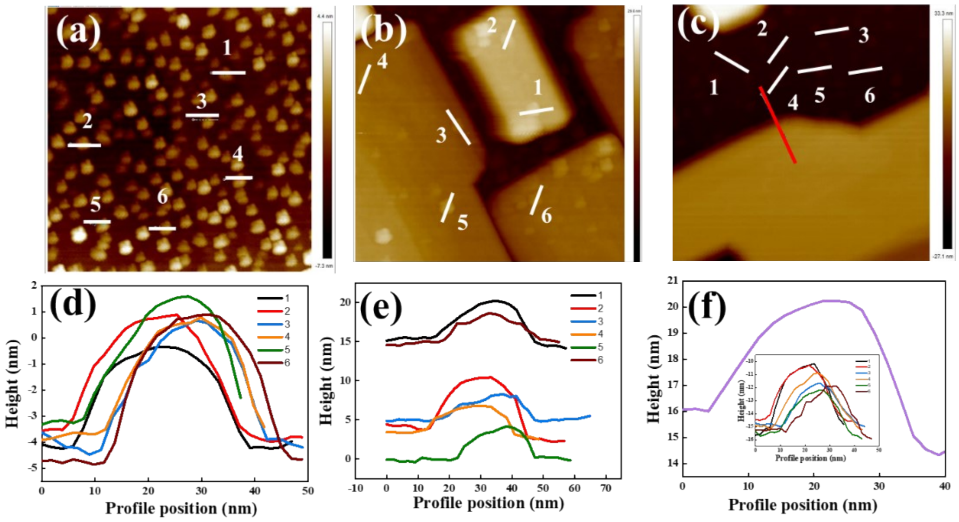

Fig. S3 The AFA images of the (a) InTe, (b) InTe (r-Te), and (c) InTe (r-In), (d-e) together with corresponding steps profile along the line.

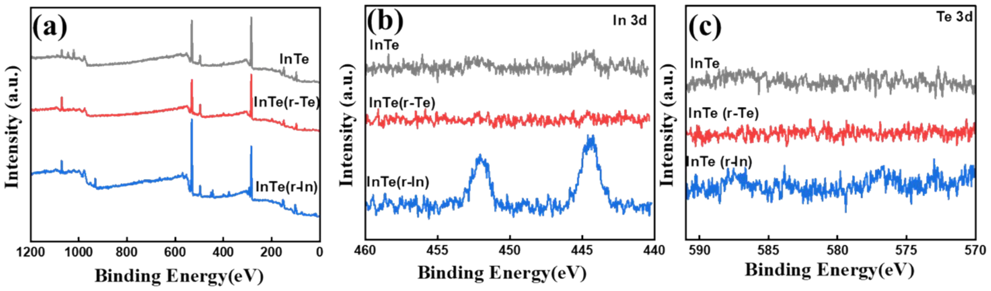

Fig. S4 (a) Survey spectra, High-resolution XPS spectra of (b) the In 3d region, (c) the Te 3d region after (b) HER and (c) OER test.

Table S1 Atomic ratio from XPS before and after OER testing.

samples	In/Te	After testing In/Te
InTe	1.437	1.25
InTe (r-Te)	0.494	1.20
InTe (r-In)	3.035	2.09

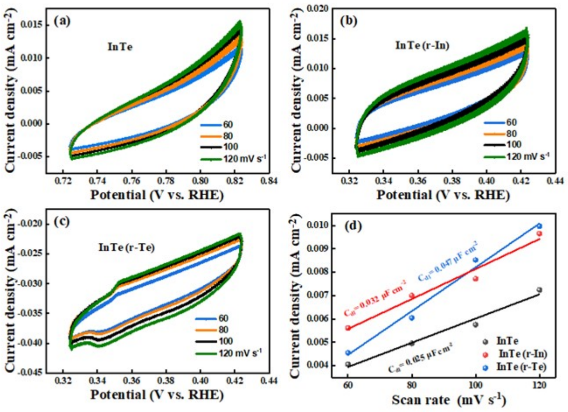

Fig. S5 The CV curves of (a) InTe, (b) InTe (r-In), and (c) InTe (r-Te) with different rates. (d) The Cdl of InTe, InTe (r-In), and InTe (r-Te).

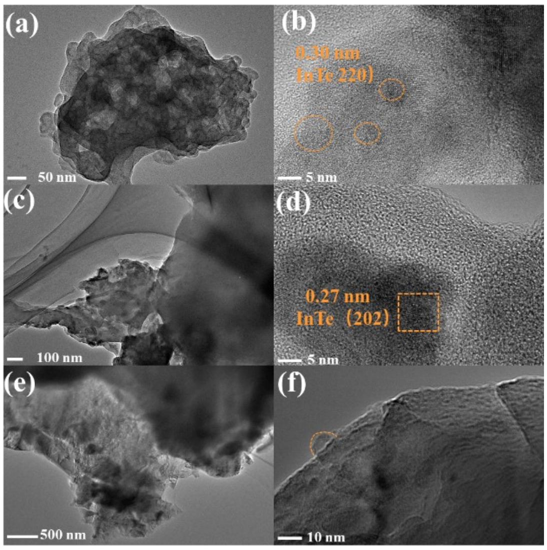

Fig. S6 The TEM and The HRTEM images of (a, b) InTe, (c, d) InTe (r-Te), and (e, f) InTe (r-In) after OER.

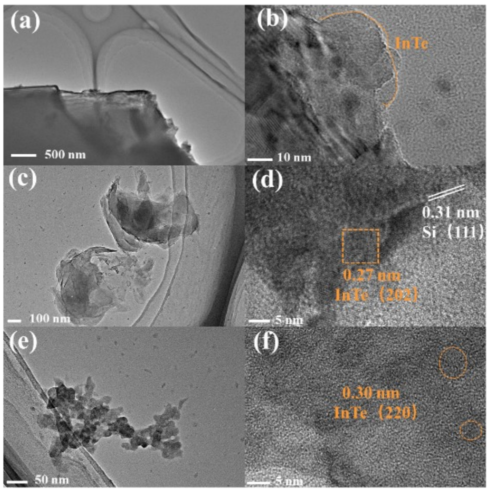

Fig. S7 The TEM and The HRTEM images of (a, b) InTe, (c, d) InTe (r-Te), and (e, f) InTe (r-In) after OER.
